# Death of Dr. James A. Gray

**Published:** 1887-10

**Authors:** 


### DR. JAMES A. GRAY IS DEAD.

This startling announcement, a few days ago, was a shock, and carried sorrow to many hearts. Not only in his native State and the city of his adoption was it felt, but by many of the profession throughout the land who knew him well, and loved him. In At- [illegible] was almost idolized by all classes of people. Especially was he held in high esteem and affectionate regard by his professional brethren : they recognized in him their peer, in every relation of life, and loved him, for his brilliant, almost transcendent attainments as a medical man, no less than for his beautiful life and character,—that of a christian gentleman ! " Pure as a pearl and as perfect," if ever truthfully written of any man, could be appropriately inscribed on the spotless tablet which shall commemorate his life,—a life gentle, and full of christian charity,—replete with deeds worthy of record above. That "death loves a shining mark," was never more fully and fearfully verified, than when it struck down this cherished, beloved man, so young, so strong and so good ; a man endeared to all who knew him. He was the friend of the poor,—the solace of the afflicted,—the counsel and companion of his collegues,—the prop and stay, the pride and hope of his aged parents, and alas, the only one on earth, (but thank God, one remains,—their faith and hope, that in the bright Beyond they shall meet their three "boys"—where partings are not known.)

Atlanta put on her weeds of deepest woe that day, and the community, as one man, mourned the loss of their favorite citizen. There was the twang of an invisible bow ; a silent arrow sped, and a Giant-heart was stilled forever ! Death is at all times terrible ; but when, with its invisible hand it strikes down by our side, our brightest and best, defying physical laws, and mocking at manhood, respecting neither the helpless loved ones, nor vigorous youth,—nor the tender, nor the aged—we tremble, as well we may ; and with heads bowed in reverential awe—exclaim "thy will be done, oh God, not mine !"

Peace to his ashes. "Green grow the turf o'er thy grave," brave, noble, generous Gray ! A stranger—one who never was blessed with even seeing him, yet, who, by correspondence, had become acquainted with his nature, and had learned to love him as a brother,—a confrere away out here in the wilds of Texas, (as the Georgia people would say), mingles his tears and regrets with those who were blessed with his friendship, cheered by his companionship, and bereaved and *impoverished* by his most untimely death!—He feels honored in the privilege of doing so !

Dr, Gray was only 37 years of age, and had practiced only ten years! Yet within that brief space he had crowded the work of a lifetime. Graduating at the Atlanta Medical College in 1877, he was, within *one year*, invited to a chair in its faculty; at one step he went to the front, and became a *leader* in the profession, whom his elders even, delighted to recognize and to honor! As managing editor of the *Atlanta Medical Journal*, he had made a name for himself, which will endure while medicine is a science, and men respect those who lend it lustre, and give to its name both dignity and honor.

